# Validation of the Waterpipe Tolerance Questionnaire among Jordanian School-Going Adolescent Waterpipe Users

**DOI:** 10.5539/gjhs.v8n2p198

**Published:** 2015-06-25

**Authors:** Sukaina Alzyoud, Sreenivas P. Veeranki, Khalid A. Kheirallah, Ali M. Shotar, Lori Pbert

**Affiliations:** 1 Faculty of Nursing, Hashemite University, Zarqa, Jordan; 2 University of Texas Medical Branch, Texas, USA; 3 Faculty of Medicine, Jordan University of Science and Technology, Irbid, Jordan; 4 Medical School, University of Massachusetts, Massachusetts, USA

**Keywords:** waterpipe, nicotine, dependence, adolescent, Jordan, validation

## Abstract

**Introduction:**

Waterpipe use among adolescents has been increasing progressively. Yet no studies were reported to assess the validity and reliability of nicotine dependence scale. The current study aims to assess the validity and reliability of an Arabic version of the modified Waterpipe Tolerance Questionnaire WTQ among school-going adolescent waterpipe users.

**Methods:**

In a cross-sectional study conducted in Jordan, information on waterpipe use among 333 school-going adolescents aged 11-18 years was obtained using the Arabic version of the WTQ. An exploratory factor analysis and correlation matrices were conducted to assess validity and reliability of the WTQ.

**Results:**

The WTQ had a 0.73 alpha of internal consistency indicating moderate level of reliability. The scale showed multidimensionality with items loading on two factors, namely waterpipe consumption and morning smoking.

**Conclusion:**

This study report nicotine dependence level among school-going adolescents who identify themselves as waterpipe users using the WTQ.

## 1. Introduction

Waterpipe use has been associated with several chronic diseases including cardiovascular disease and cancer (Akl et al., 2010; World Health Organization [WHO], 2005). A major concern of waterpipe use is the dependence on nicotine resulting from tobacco, the main substance used in the waterpipe (Kandel, Hu, & Griesler, 2007; O’Loughlin, DiFranza, & Tarasuk, 2002). Several measurement tools have been used to assess nicotine dependence level among tobacco users and one such tool that has been widely used is the modified Fagerstrom Tolerance Questionnaire (mFTQ) (Prokhorov, Pallonen, Fava, & Ding Niaura, 1996; Salameh, Waked, & Aoun, 2008). Previous studies have reported the reliability and validity of the mFTQ among adult cigarette smokers (Burling & Burling, 2003; Prokhorov, Pallonen, Fava, & Ding Niaura, 1996; Prokhorov, Koehly, Pallonen, & Hudmon, 1998), adolescent cigarette smokers (Prokhorov et al., 2001; Rojas, Killen, Haydel, & Robinson, 1998) and adult waterpipe users (Auf et al., 2012). However, research is limited in understanding nicotine dependence among adolescent waterpipe users (Primack et al., 2014), and to our knowledge no study has been conducted to estimate nicotine dependence among school-going adolescent waterpipe users.

Earlier studies have demonstrated that the onset of smoking typically begins before or during adolescence and potentiates an individual to become a regular smoker in adulthood (Audrain-McGovern et al., 2004; Chassin, Presson, Rose, & Sherman, 1996; Karp, O’Loughlin, Paradis, Hanley, & DiFranza, 2005). Tobacco use, which includes cigarette smoking, use of alternate or non-cigarette tobacco products and Environmental Tobacco Smoke (ETS) exposure, among adolescents has been on the rise in the low- and middle-income countries including Jordan, in which 21% of Jordanian adolescents aged 13 to 15 currently smoke waterpipe (Alzyoud, Weglicki, Kheirallah, Haddad, & Alhawamdeh, 2013; A. Boutayeb & S. Boutayeb, 2005; Center for Disease Control [CDC], 2010; Warren, Jones, Eriksen, & Asma, 2006). Waterpipe use is the form of tobacco use that has been rapidly increasing and gaining significant attention among youth in Arab nations, and this is particularly evident among girls (Alzyoud et al., 2013, 2014; Dar-Odeh & Abu-Hammad, 2011; Mzayek et al., 2012). Previous studies have reported that smoking cigarettes creates nicotine dependence and potentiates transformation into regular cigarette smoking and addiction (Carpenter, Baker, Gray, & Upadhyaya, 2010; Kandel et al., 2007; Nonnemaker & Homsi, 2007; Savageau, Mowery, & DiFranza, 2009). In addition, previous researchers have demonstrated a link between nicotine intake and development of dependence among adolescents (Russell, 1980; Rubinstine, Tompson, Benoweitz, Shiffman, & Moscicki, 2005). Russell (1980) has theorized that nicotine tolerance will result in increased intake that will further lead to dependence. Rubinstine et al., (2005) reported that adolescents had higher cotinine level earlier in the morning as a result of cravings for the first dose of nicotine, and it also depends on the smoking patterns of adolescents. However, there is a lack of standardized scale to measure the level of nicotine dependence among adolescents. Researchers have developed a scale that assessed nicotine dependence among adult waterpipe users (Primack et al., 2014; Salameh et al., 2008). The scale, Lebanese Waterpipe Dependence Scale (LWDS-11), was originally developed by Salameh and colleagues (2008) to estimate waterpipe dependence among adults (Salameh et al., 2008), and further utilized to determine nicotine dependence among waterpipe smoking college-going students (Primack et al., 2014). It should be emphasized that both (Primack et al., 2014; Salameh et al., 2008) were conducted among the adult population, while the current study is to validate a nicotine dependence scale among adolescent.

As waterpipe use is becoming more prevalent among the youth population in Jordan and other neighboring countries in the Middle East (Alzyoud et al., 2014; Akl et al., 2013; Al-Lawati, Muula & Hilmi, 2008) we found that studies that reported valid and reliable scale measuring nicotine dependence among Arab youth waterpipe users were not identified. The purpose of the current study was to assess the reliability and validity of an Arabic version of the mFTQ adapted for waterpipe users, the modified Waterpipe Tolerance Questionnaire (WTQ), among a sample of Jordanian adolescents aged 11 - 18 years old. The study received institutional review board approval from The Hashemite University Institutional Review Board and Zarqa Governorate Educational Districts.

## 2. Methodology

### 2.1 Study Design, Setting and Population

A cross-sectional study was conducted among a sample of school students in one of Jordan’s major governorates. The study aimed to assess the validity and reliability of the modified Waterpipe Tolerance Questionnaire (WTQ) among waterpipe users. The current study was conducted in the public schools of Zarqa Governorate. A this governorate along with Amman and Balqa’ governorate represents the central region of Jordan. Zarqa ranks as the third most densely populated governorate in Jordan (DOS, 2012), which represents 15% (total population of 931,100) of Jordan’s total population (DOS, 2012).

**Figure 1:**
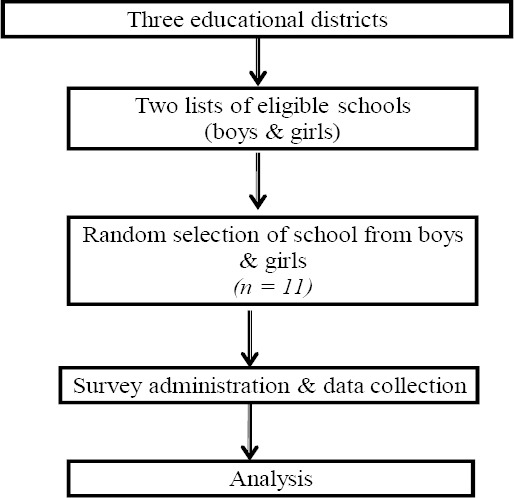
Study partipants process of recriuitment

Zarqa governorate is divided into three educational districts (First, Second, and Al-Rusaefah) Figure 1 show the process of participants recruitment. These districts hold a total of 225 basic level (i.e., grades 1 - 10, ages of 6 - 16 years) and 109 high level grade schools (i.e., grades 11-12, ages of 17-18 years) that are distributed across the three districts. The total number of students enrolled in all the schools is estimated to be 219,701, with 108,933 girls and 110,768 boys (DOS, 2014). However, only schools that included students in grades 6^th^-12^th^, which met study sample desired age, were invited to participate in the study. Students in Jordan’s public schools are segregated into boys’ and girls’ schools. The final sample for the current study included 333 school students aged 11 - 18 years, and had previously used waterpipe.

### 2.2 Recruitment, Informed Consent, and Data Collection

After obtaining approvals from the Institutional Review Board of the Hashemite University (HU) and Zarqa Governorate Three Educational Districts, the Principals of each selected school were approached to obtain their verbal permission to conduct the study and access the students. Participating students were provided with an explanation of the study purpose and objectives, and were provided an opportunity to ask any question or concern related to the study. Participants were asked to provide their written informed consent prior to completing study questionnaires.

The participant selection procedure used a multistage random sampling technique to recruit students from the three Educational Districts. The sampling procedure was conducted by using lists of schools from each of the three Educational Districts. To recruit a representative sample of boys and girls, two lists were used, one for boys’ and one for girls’ schools. Each of these lists contained both schools in basic and high school educational levels that had 6^th^ grade or higher grade level. Using systematic random sampling technique where every 5^th^ school was randomly selected, the desired number of schools (11 schools) was drawn. In the final stage, selection of classrooms was also randomly done from each selected school and all students in the selected classrooms were eligible to participate in the study. Overall a total of 1,050 school students were invited to participate in the study, of which 95.2% completed the questionnaires. Three hundred thirty three students self-identified themselves as waterpipe users and were included in the final study sample.

### 2.3 Measures

Background information including age, grade, and gender was obtained using a demographic information questionnaire. Other measures included participants’ daily cigarette smoking, daily waterpipe use (defined as use of waterpipe more than once during the day), nicotine dependence (ascertained using *Waterpipe Tolerance Questionnaire* (*WTQ*)), religion, and school academic performance (measured using GPA scored during the previous academic year).

An adaptation of the modified Fagerstrom Tolerance Questionnaire (mFTQ; Prokhorov et al., 1996), called the Waterpipe Tolerance Questionnaire (WTQ), was used to measure self-reported level of nicotine dependence among waterpipe users. The mFTQ was modified by replacing the words “cigarette” with “waterpipe” in the original questionnaire. The responses were also modified to be more reflective of waterpipe smoking. Only participants who self-reported themselves as waterpipe users were instructed to respond the WTQ. The final questionnaire included six questions for waterpipe users. The original mFTQ developed by Prokhorov and colleagues (1996) for adolescent smokers consists of a seven item questionnaire, however one question, namely “which cigarette would you hate to give up?” was not included in the WTQ as this question was not applicable to waterpipe users. The remaining six items were administered to waterpipe users by replacing “cigarette” with “waterpipe” in the question. Similarly we tailored the responses to be applicable to the tobacco users in Jordan (see Table 1). We standardized the scoring of the dependence level to minimize differences between the WTQ instruments used in cigarette smokers and waterpipe users and also to compare individual items effectively. Each individual item question in the WTQ was measured using Likert-type responses. The maximum score of the nicotine dependence level is eight. We categorized the scores into low (score 0-1), moderate (score 2-3) and high (score 4-8) dependence levels by tertiles.

**Table 1:** Waterpipe Tolerance Questionnaire (WTQ): survey items, response and scoring of nicotine dependence level among Jordanian school-going adolescent waterpipe users

Question	Response	Scoring
How many times a day (if at all) do you smoke waterpipe?	less than ½ head or none Half head to one head/day More than one head /day	0 1 2
If you smoke waterpipe, do you inhale when you smoke?	Never Seldom or quite often Always	0 1 2
How soon after you wake up do you smoke your first waterpipe?	More than 30 minutes after waking Within the first 30 minutes	0 1
Do you find it difficult to refrain from using tobacco in places where it is forbidden (e.g. Mosque, Church, library, school, movies?	No Yes	0 1
Do you use tobaccomore frequently during the first hours after waking up than you do during the rest of the day?	No Yes	0 1
Do you use tobacco more during the first 2 hours after waking up than during the rest of the day?	No Yes	0 1

Maximum score		8

### 2.4 Statistical Analysis

We analyzed the adolescent waterpipe users’ sub-sample of data separately. To assess the internal validity of WTQ for adolescent waterpipe users, we conducted exploratory factor analysis using principal-component analysis. We examined eigen values, scree plot, and the number of factors to be included in the final score. We then re-conducted principal-component analysis while forcing this number of factors by adjusting for the factor weights. We provided information concerning the allocation of the individual items to any dimensions of the WTQ for waterpipe users. We computed cronbach’s alpha to test for internal consistency. We reported correlation for the overall WTQ and its individual items for adolescent waterpipe users. In addition, to assess construct validity we tested for association of nicotine dependence (categorized into three groups) with waterpipe use related characteristics, include age of initiation of waterpipe (categorized into 10 years and younger, 11-13, 14 years and older), number of heads smoked per day in the past month, number of sessions of waterpipe used in the past week, respectively using chi-square, Cramer V statistic contingency statistic test. A two-tailed alpha of 0.05 was considered statistically significant. Data was managed and analyzed using SAS version 9.3 (SAS Institute Inc., Cary, NC).

## 3. Results

Of the 1,050 adolescents who were provided questionnaires, 993 responded while 57 did not and were not included in the study. Since the primary focus of the current study was to understand nicotine dependence among waterpipe users, the final sample included adolescents who self-identified themselves as waterpipe users and provided complete information on the questionnaires resulting in a final sample of 333 waterpipe users. Table 2 presents characteristics of the study participants. The results indicated that approximately 2/3 of the sample included girls and (approximately one-third included adolescents aged 16 years. Forty seven percent of participants were studying 10^th^ grade, and 32.7% received good GPA in the previous year.

**Table 2:** Demographic characteristics of adolescent waterpipe users

Characteristic	N	%
**Age**		
11	9	2.70
12	21	6.31
13	35	10.51
14	24	7.21
15	55	16.52
16	102	30.63
17	87	26.13
**Sex**		
Male	111	33.33
Female	222	66.67
		
**Religion**		
Muslim	305	91.59
Crust	28	8.41
**Grade**		
6^th^	30	9.01
8^th^	59	17.72
10^th^	157	47.15
12^th^	87	26.13
**Grades in last year**		
Excellent(90-100)%	47	14.11
Very good(80-89)%	85	25.53
Good(70-79)%	109	32.73
Poor (69% or less)	92	27.63

An exploratory analysis using principal components analysis was conducted to test for internal validity of the WTQ among waterpipe users. Principle components analysis with the six WTQ items yielded two eigen values >1.0 and a scree plot suggesting a two-factor solution. Upon forcing the two-factor solution to standardize the loadings, we found that two items in the WTQ for waterpipe users loaded strongly on factor 1, one item loaded moderately on factor 1 and two items loaded strongly on factor 2, respectively. From the six items, one item about how soon a person smokes his/her waterpipe after waking up was eliminated because it did not uniquely load on either of the factors. The results indicated that the scale is two dimensional with three items loading on factor 1, *Waterpipe Consumption*, and two items loading on factor 2, *Morning Smoking* (Table 3).

**Table 3:** Exploratory factor analysis: Factor structure for dependence items

Item	Factor1	Factor 2
Number of waterpipes smoked per day		0.70378
Inhale while smoking waterpipe	0.54591	
Difficult to refrain from using tobacco in places where it is forbidden		0.76197
Smoke waterpipe more during the first hours than you do during the rest of the day	0.89112	
Tobacco use during first 2 hours of waking		0.87621

In addition, we tested for scale reliability with the current sample. Cronbach’s alpha coefficients are satisfactory, ranging from moderate (α = 0.53) to high (α= 0.80) correlation for individual items, and 0.73 for the total scale. Table 4 presents the correlations for the overall WTQ scale and its individual items. It was found that the overall WTQ scale correlated significantly with every individual item with high correlation with smoking waterpipe during the first hours after awake. High correlation was found between adolescent waterpipe use during the first hours after waking up and the use of tobacco during the first two hours of waking up (r = 0.77133; p < 0.0001). In addition, a significant correlation was found between inhaling while smoking waterpipe and difficulty to refrain from using waterpipe in forbidden places (r= 0.1944, p<0.0001), smoking waterpipe during the first hours after waking up, and the use of tobacco during the first 2 hours of waking up (r= 0.7713, p < 0.0001). Using the WTQ that consisted of five individual items, we scored the responses of waterpipe users and found that the overall mean score for WTQ five item scale was 2.65 [Standard Deviation (SD) 1.62], with mean scores for individual items ranging from 0.14 (SD 0.46) to 1.27 (SD 0.77).

**Table 4:** Correlations between individual items for the FTQ for waterpipe using adolescents

Item	Number of waterpipes smoked per day	Inhale while smoking water pipe	Difficult to refrain from using tobacco in places where it is forbidden	Smoke waterpipe more during the first hours after waking up than the rest of the day	Tobacco use during first 2 hours of waking
**Overall score**	0.26903	0.70439	0.48380	0.72656	0.72222
Number of water pipes smoked per day	1.00000	-0.10396	0.10624	-0.00264	-0.00083
Inhale while smoking water pipe		1.00000	0.19435	0.34081	0.31940
Difficult to refrain from using tobacco in places where it is forbidden			1.00000	0.08432	0.10189
Smoke water pipe during the first hours after waking up than you do during the rest of the day				1.00000	0.77133
Tobacco use during first 2 hours of waking					1.00000

We categorized the nicotine dependence level scores into tertiles, and found that approximately 27%, 38.4%, and 34.4% of school-going adolescent waterpipe users had low, moderate and high level of nicotine dependence, respectively. Table 5 presents the differences in adolescents’ nicotine dependence categories by selected waterpipe use characteristics including age of initiation of waterpipe use, number of heads smoked per day and frequency of sessions per week. Significant differences in adolescents’ nicotine dependence were identified by selected waterpipe use characteristics (p<0.0001), with a majority of adolescents with high nicotine dependence level smoking more than four heads per day (54.2%) and >10 sessions per week (57.9%).

**Table 5:** Association between waterpipe use-related characteristics and nicotine dependence among adolescent waterpipe users

		Nicotine dependence among waterpipe users				

	All participants	Low; 89 (26.7)	Moderate; 128 (38.4%)	High; 116 (34.8%)	

Waterpipe use characteristics	N (%)	%	%	%	p-value
Age of initiation				
8-10 years	36(10.81)	16.7	30.6	52.8	0.07
11-13 years	102(30.63)	14.7	37.3	48.0	
14-16years	58(17.42	24.1	48.3	27.6	
Heads smoked per day ***					<0.0001
≤ 1	219(65.77)	34.2	39.3	26.5	
2-3	86(25.83)	12.8	34.9	52.3	
≥ 4	24(7.21)	4.2	41.7	54.2	
Sessions per week ***					<0.0001
≤ 2	107(32.13)	49.5	43.9	6.5	
3-9	78(23.42)	25.6	43.6	30.8	
≥ 10	145(43.54)	10.3	31.7	57.9	
Sex**					
Male	111 (33.33)	34.2	43.2	22.5	0.003
Female	222 (66.67)	23.0	36.0	41.0	

*p<0.05,

** p<0.01,

*** p<0.001.

## 4. Discussion

The current study demonstrated that a large percentage of the study sample had used waterpipe. The study also demonstrated that the WTQ could be used to measure nicotine dependence among school-going adolescents who currently used waterpipe. The data suggested a 5-item scale with two-factor structure representing “waterpipe consumption” and “morning smoking” as key components of the WTQ. Additionally, as evidenced by associations between the WTQ and study variables, the overall scale seems to have good reliability and internal validity in this population. Our results for reliability were consistent with previous findings of the mFTQ internal consistency (Prokhorov et al., 1996). These results indicate that the WTQ can be used by researchers to measure nicotine dependence among adolescent waterpipe users. This study report adapting the mFTQ to be used with school-going adolescent waterpipe users. Additionally, it is the first study to report levels of nicotine dependence among Jordanian school-going children who are waterpipe users.

In their study to validate the Lebanon Waterpipe Dependence Scale (LWDS-11) Salameh and colleagues (2008) reported that items relative to morning waterpipe smoking were removed from the analysis because they did not correlate with the other items. However, in our study items related to morning smoking loaded uniquely on factor 1, which possibly indicate that participants may take the trouble of preparing the waterpipe in the morning to satisfy their cravings for nicotine. Studies have shown that tobacco users might smoke in the morning as a result of experiencing craving symptoms that stem from being dependent on nicotine (Nosen, & Woody, 2013; Zhou et al., 2009). Our results are consistent with previous studies using the FTQ among adolescent cigarette smokers (Cohen, Myers, & Kelly, 2002; Wellman et al., 2006). The results indicated that the inhalation question added value to the WFTQ. This result may indicate that participants might need to inhale the smoke to gain a dose of nicotine. This result, however, is inconsistent with the previous study of tobacco smokers using the FTQ revised version, where this question did not load uniquely on any factors and therefore was removed from the analysis (Heatherton, Kozlowski, Frecker, & Fagerström, 1991). However their study was conducted among adult cigarette smokers. The distinction between our study and the Heatherton et al. study could be explained by the difference between adolescent and adults’ susceptibility to developing dependence on nicotine. O’Loughlin and colleagues (2002) suggested that adolescent tobacco users may show signs of dependence even when smoking rates are low and sporadic. Moreover, when in use, waterpipes produce more smoke than cigarettes that linger in the surrounded atmosphere which makes it difficult for the smoker not to inhale it. The other factor could be related to the appealing scent of the smoke which comes from the added flavors to the tobacco used in the waterpipe head.

A large proportion of study participants had a score of moderate or high level of nicotine dependence indicating that adolescent use of waterpipe significantly exposes them to being dependent to nicotine. Moreover, a significant difference was found between levels of nicotine dependence and number of waterpipe use sessions per week and number of waterpipe heads. These results indicated that the largest proportion of participants who had a moderate level of nicotine dependence smoked more than one session and head of waterpipe. Research has suggested that use of waterpipe once each day results the blood nicotine concentration of someone who smokes 10 cigarettes daily (Shafagoj, Mohammed, & Hadidi, 2002). Another study reported that a single waterpipe session is associated with the nicotine content in about 2-3 cigarettes (Neergaard, Singh, Job, & Montgomery, 2007). Another possible reason for the finding of moderate to high nicotine dependence among the sample of adolescent waterpipe users is the finding from a number of studies that initial symptoms of dependence for adolescents appear soon after smoking initiation (Difranza, 2008; Kandel et al., 2007; Savageau et al., 2009). These studies support our findings that a large proportion of participants who initiated waterpipe use at age 8 to 10 had a high level of nicotine dependence.

Our study has strength of high response rate from the participants. This study also includes limitations that merit discussion. Only a descriptive self-reporting scale was used to measure nicotine dependence level among adolescents, and future studies are needed for an objective measurement of nicotine level. Exploratory factor analysis was used to provide evidence for construct validity, however confirmatory factor analysis was not conducted due to low sample size, and future studies should include such analysis with large sample size. WTQ was developed by replacing the words “cigarette or tobacco” in the mFTQ to “waterpipe use”, and this definition of assessing nicotine dependence level among waterpipe users is yet under discussion. Another limitation is that the findings should not be generalized to the general population of Jordanian adolescents since the data only reflect findings from one governorate in Jordan. Moreover, testing the possible association between nicotine dependence levels and use of cigarette, waterpipe and/or dual use is beyond the scope of the current study, and future research should be conducted to assess such relationship. Lastly, the study is cross-sectional in nature and limits causal inferences.

In conclusion, our study demonstrated that the WTQ is a reliable instrument that can be used among adolescent waterpipe users to assess the level of nicotine dependence. In addition, study findings reported that the largest proportion of adolescent who used waterpipe had either moderate or high level of nicotine dependence. These findings illustrate the need to educate health care professionals such as the school nurses about the threat of the emerging epidemic of waterpipe use among youth. Being in constant and direct contact with adolescents in the school the nurses have an advantage of assessing nicotine dependence among this population. School nurses could play vital role in developing and implementing waterpipe use prevention programs that targets school students in order to limit their use and interest of waterpipe.
